# Inflammatory pseudotumor-like follicular/fibroblastic dendritic cell sarcoma: focus on immunohistochemical profile and association with Epstein-Barr virus

**DOI:** 10.1186/s13027-022-00474-8

**Published:** 2022-12-24

**Authors:** Francesca Pagliuca, Andrea Ronchi, Annamaria Auricchio, Eva Lieto, Renato Franco

**Affiliations:** 1 Pathology Unit, Department of Mental and Physical Health and Preventive Medicine, University of Campania “Luigi Vanvitelli”, Via Luciano Armanni 5, 80100 Naples, Italy; 2 Division of Gastrointestinal Tract Surgical Oncology, Department of Translational Medical Sciences, University of Campania ‘Luigi Vanvitelli’, 80138 Naples, Italy

**Keywords:** Inflammatory pseudotumour-like follicular/fibroblastic dendritic cell sarcoma, Epstein-Barr virus, Immunohistochemistry

## Abstract

Inflammatory pseudotumour-like follicular/fibroblastic dendritic cell sarcoma (IPT-like FDCS) is a rare EBV-associated variant of follicular dendritic cell sarcoma, usually arising in the liver or spleen and characterized by a favourable prognosis. The neoplastic cells show variable follicular dendritic cell or fibroblastic reticular cell differentiation and their immunoprofile is still poorly characterized. We describe a case of splenic IPT-like FDCS with unexpected CD31 expression and provide a concise review of English literature on the topic.

## Introduction

Follicular dendritic cell sarcoma (FDCS) is a well-characterised neoplastic proliferation of follicular dendritic cells arising in lymph nodes or in extranodal sites, including tonsils, gastrointestinal tract, mediastinum, peritoneum and lung. However, a subgroup of FDCSs, usually affecting the spleen and the liver, shows distinctive clinical, histomorphological and molecular features, such as marked female predilection, indolent clinical course, presence of a prominent inflammatory background and association with Epstein-Barr virus (EBV). Due to the intense inflammatory infiltrate, these tumours can morphologically mimic an inflammatory pseudotumour (IPT) or an inflammatory myofibroblastic tumour (IMT). In the latest WHO classification of Haematopoietic and Lymphoid Tissue Tumours [1], this peculiar form of FDCS is recognised as a separate variant and named IPT-like follicular/fibroblastic dendritic cell sarcoma. Such definition reflects the inconstant immunophenotype of the neoplastic cell population, showing variable follicular dendritic cell or fibroblastic reticular cell differentiation. Herein we describe a case diagnosed as splenic IPT-like FDCS with peculiar immunohistochemical findings.

## Case presentation

**Fig. 1:**
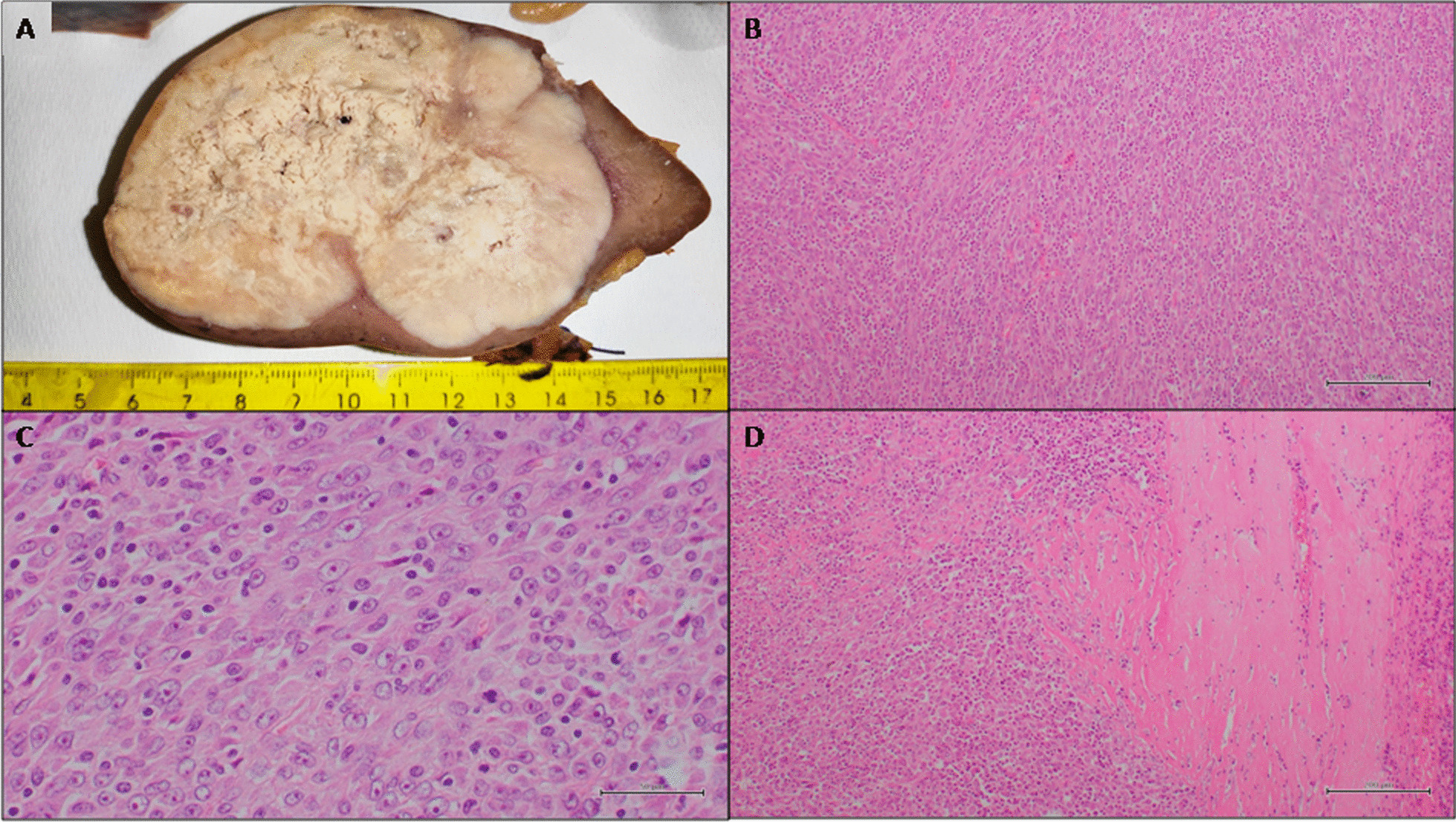
Gross and histopathological findings. **A **Gross appearance of the splenic lesion on sectioning. **B** Microscopic appearance of the lesion: intersecting fascicles of spindle-to-ovoid cells within an inflammatory background (Hematoxylin and Eosin stain; original magnification: 100x). **C **On higher magnification, neoplastic cells show large, ovoid nuclei with prominent eosinophilic nucleoli; some mitoses are identified (Hematoxylin and Eosin stain; original magnification: 400x). **D **Areas of hyaline fibrosis with plasma cell infiltration (Hematoxylin and Eosin stain; original magnification: 100x)

**Fig. 2:**
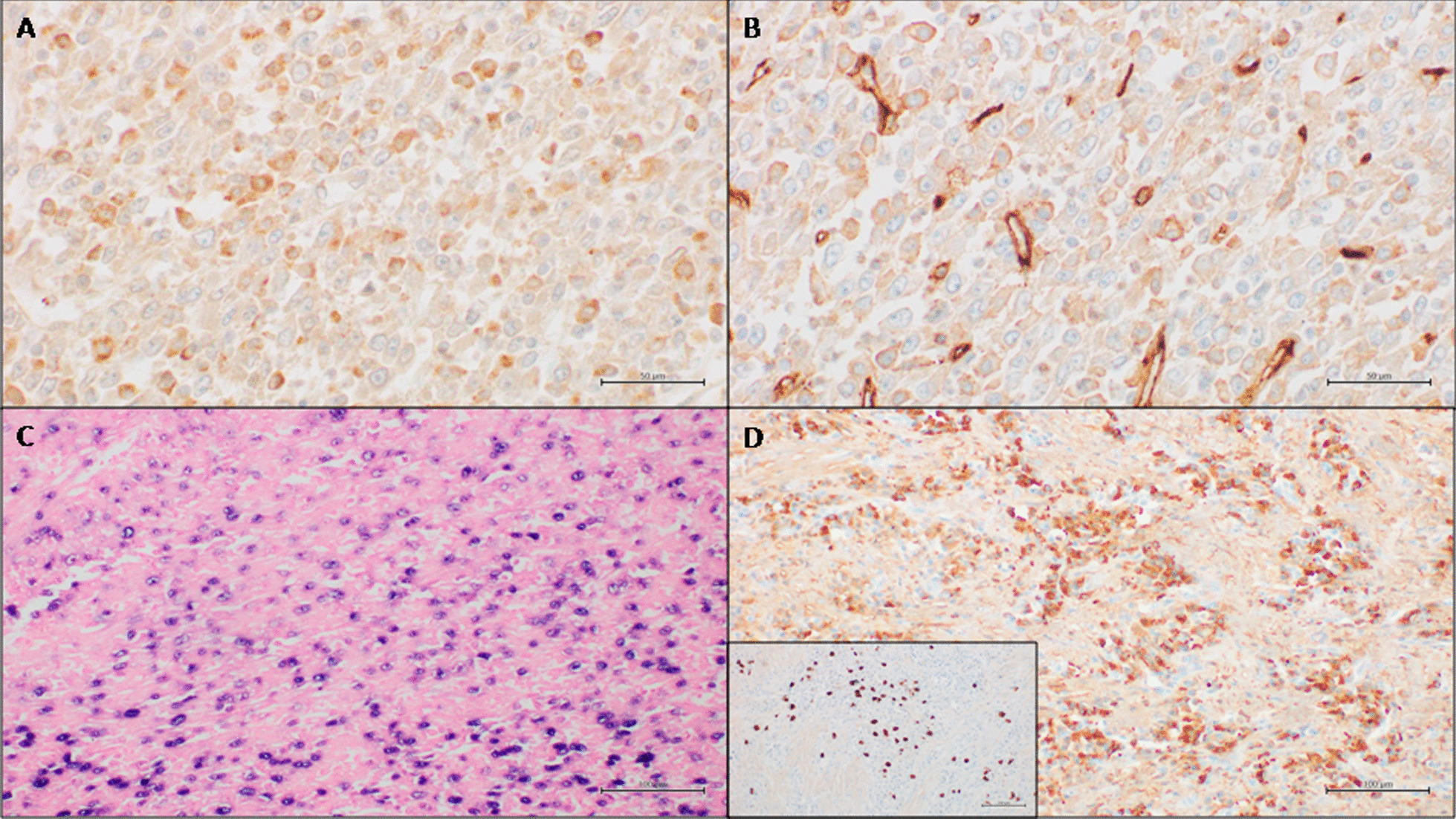
Immunohistochemical and molecular findings. **A** Faint CD68 expression in neoplastic cells (CD68 immunostain; original magnification: 600x). **B** Faint CD31 expression in neoplastic cells (CD31 immunostain; original magnification: 600x). **C** Epstein-Barr virus-encoded RNA is detected in neoplastic cells (EBV chromogenic in situ hybridization; original magnification: 200x). **D** IgG-positive plasma cells in large fibrotic areas (IgG immunostain; original magnification 200x). Shown as inset: approximately 25% of IgG-positive plasma cells are IgG4-positive (IgG4 immunostain; original magnification: 200x)

The patient was a 63 year-old female with well-controlled type 2 diabetes mellitus presenting with rapid and unexplained weight loss. Past medical history was unremarkable. Laboratory tests only highlighted a mild anemia (hemoglobin: 9,7 g/dL). Total-body computed tomography revealed the presence of a large, solitary splenic mass with inhomogeneous density and peripheral enhancement interpreted as a primitive neoplastic process. The patient underwent a splenectomy with associated removal of splenic hilar lymph nodes. On gross examination, the spleen was moderately enlarged, with a smooth surface and intact capsule. It measured cm 15 × 12 × 6,7 and weighted g 557. On gross sectioning, it was almost entirely occupied by a gray-whitish solid mass with central necrosis (Fig. 1A). Microscopic examination showed a well-circumscribed, unencapsulated proliferation of ovoid, spindle or stellate cells arranged in interlacing fascicles within an inflammatory background (Fig. 1B). The cells were characterized by a mild pleomorphism, oval vesicular nuclei with occasionally prominent nucleoli, conferring a Hodgkin cell-like appearance; cytoplasms were eosinophilic, with indistinct contours. Hyperchromatic and degenerated cells were also seen, reminiscent of mummified cells in Hodgkin lymphoma. Mitotic index was 5–10/10 hpf (Fig. 1C). The admixed inflammatory cells included lymphocytes, plasma cells, neutrophils and eosinophils. Large fibrotic areas with high plasma cell density and areas of necrosis were noted. Immunohistochemical analysis demonstrated faint expression of CD68 and CD31 (Fig. 2A, B) in a fraction of tumour cells (approximately 70% and 30% of all tumour cells, respectively); smooth muscle actin (SMA) was positive only in isolated cells; CD21, CD23, CD45 (LCA), CD34, ERG, factor VIII, podoplanin (D2-40), S100, desmin, calponin, h-caldesmon, ALK and CD1a were consistently negative. EBER (EBV-encoded small RNAs) in situ hybridization showed a positive nuclear result in the spindle/ovoid cells whereas inflammatory cells and cells in the surrounding splenic parenchyma were negative (Fig. 2C). The plasma cell population in the large fibrotic areas resulted IgG-positive, with an IgG4/IgG ratio of approximately 25% (Fig. 2D). Hilar lymph nodes were morphologically unaltered. A final diagnosis of pseudotumour-like follicular/fibroblastic dendritic cell sarcoma limited to the spleen with tumor-free margins was rendered.

## Discussion

IPT-like FDCS was firstly described as a distinctive variant of FDCS in 2001 [2] but, to the best of our knowledge, the very first reported case dates back to 1996 [3]. Since then, approximately 50 cases have been reported in literature under such definition [4, 5]. The main differences distinguishing IPT-like FDCS from conventional FDCS encompass a striking female predominance, selective intra-abdominal localization, association with EBV and excellent outcome in most cases after local excision.

Epstein-Barr virus (EBV) has been increasingly recognised to be involved in the development of a range of neoplastic and pseudo-neoplastic diseases, including both haematolymphoid and epithelial tumours. EBV-associated tumours comprise, among others, Burkitt lymphoma, Hodgkin lymphoma, post-transplant lymphoproliferative disorders, nasopharyngeal carcinomas, gastric and breast carcinomas and smooth muscle cell-derived tumours in immunodeficient individuals.

The exact mechanisms of EBV-related oncogenesis have partially been elucidated. Viral genome integrates into the genome of the host cell, establishing a latent infection, and the expression of viral products drives cell proliferation.

By definition, the demonstration of EBV in the neoplastic population, usually achieved by EBV-encoded RNA (EBER) in-situ hybridization (ISH) or immunohistochemistry (IHC) for latent membrane protein, is the key to the diagnosis of IPT-like FDCS.

IPT-like FDCS has been almost exclusively described in liver and spleen, with only two cases presenting as colonic polypoid masses [6, 7] and two cases involving the pancreas [8] and peri-pancreatic soft tissues [2]. Histologically, tumour cells are sparsely distributed within a prominent inflammatory infiltrate, mainly comprised of lymphocytes and plasma cells, not usually seen in FDCS. Occasionally, variable amounts of eosinophils and histiocytes can also be present. Neoplastic cells show a variable degree of nuclear atypia, which is often minimal to mild. Tracing the line between IPT-like FDCS, inflammatory pseudotumour (IPT) and inflammatory myofibroblastic tumour (IMT) may be impossible on a morphological basis alone and definitely requires immunohistochemical/molecular studies. It is likely that lesions variably classified in the past as IPT, IMT or IPT-like FDCS actually represent the same entity or, at least, part of a unique spectrum, as suggested by similarities in clinical, radiological and morphological findings [9]. The neoplastic nature of the lesion is confirmed by the occurrence, albeit rare, of local recurrences and aggressive clinical course as well as by studies indicating EBV monoclonality in tumour cells [10].

The exact derivation of IPT-like FDCS is debated and still unclear. In the latest WHO classification [1], it has newly been designated as ‘inflammatory pseudotumour-like follicular/fibroblastic dendritic cell sarcoma’, highlighting the uncertain phenotype of the cell of origin. In fact, in contrast to FDCSs, IPT-like FDCSs show inconstant expression of follicular dendritic cell (FDC) markers, including CD21, CD23 and CD35; moreover, they may express SMA, CD68 and/or desmin, pointing towards a fibroblastic reticular cell (FRC) differentiation. Of notice, several studies have demonstrated a common origin for FDCs and FRCs, with divergent differentiation from the same progenitor.

Recently introduced FDC markers including fascin, clusterin, gamma-synuclein, D2-40 and others are not commonly available for diagnostic purposes and their sensitivity/specificity for a diagnosis of FDCS is still poorly defined. As a consequence, the absence of FDC marker expression can no longer exclude a diagnosis of IPT-like FDCS.

Currently, the mainstay to pose such diagnosis reasonably is the demonstration of EBV in a coherent clinical-morphological context.

In the present case, we failed to demonstrate a FDC-like immunoprofile; CD68 and partial SMA positivity could instead indicate a FRC differentiation. One interesting finding is the expression of the endothelial marker CD31. The majority of cases previously reported as IPT-like FDCS did not mention any result for CD31 staining and it is likely that CD31 was not included in the ordered immunohistochemical panel. Therefore, the frequency of CD31 expression in IPT-like FDCS cannot be accurately established. After review of the literature, we could find only another case of IPT-like FDCS with reported CD31 expression [11]. However, in this case, concomitant expression of CD21 and CD23 corroborated the diagnosis. Endothelial cells (ECs) are also part of non-haematopoietic stromal cells in secondary lymphoid organs and thus share a common origin with FDCs and FRCs. Arguably, this could explain the aberrant expression of CD31 in FDC/FRC-derived neoplasms. CD31 expression in splenic IPT-like FDCS may represent a diagnostic pitfall as vascular tumors are commonly encountered non-lymphoid neoplasms in the spleen.

An intriguing association between IPT-like FDCS and IgG4-related disease has recently emerged. In a retrospective Korean study, Choe et al. [12] described six cases of splenic IPT-like FDCSs, all characterized by heavy IgG4 + plasma cell infiltration. The authors propose a link between immune dysregulation caused by EBV, IgG4 + plasma cell activation and the development of the neoplasm. Our case showed areas of hyaline fibrosis with high plasma cell density. Plasma cells were of the IgG type with a substantial proportion of IgG4 + elements. However, no data about serum IgG4 levels in our patient were available.

Apart from the presence of monoclonal EBV gene, the genomic landscape of IPT-like FDCS remains largely undefined. In a recent paper, comprehensive next-generation sequencing analysis on two cases of IPT-like FDCSs failed to demonstrate any pathogenic variant of potential or strong clinical significance within the targeted regions of the evaluated genes [13].

## Conclusion

In conclusion, IPT-like FDCS is a rare but likely under-recognised EBV-related tumour of unclear derivation characterized by good clinical outcome, mainly found in spleen and liver. Detection of EBV plays a critical role to reach the correct diagnosis because the immunohistochemical profile of tumour cells is extremely variable.

## Data Availability

Not applicable.

## Abbreviations

IPT-like FDCS: inflammatory pseudotumour-like follicular/fibroblastic dendritic cell sarcoma
FDCS: follicular dendritic cell sarcoma
EBV: Epstein-Barr virus
IPT: inflammatory pseudotumour
IMT: inflammatory myofibroblastic tumour
WHO: World Health Organization
EBER: EBV-encoded small RNAs
SMA: smooth muscle actin
FDC: follicular dendritic cell
FRC: fibroblastic reticular cell
